# Spatial clustering of heroin-related overdose incidents: a case study in Cincinnati, Ohio

**DOI:** 10.1186/s12889-022-13557-3

**Published:** 2022-06-25

**Authors:** Jung Im Choi, Jinha Lee, Arthur B. Yeh, Qizhen Lan, Hyojung Kang

**Affiliations:** 1 Data Science, Bowling Green State University, 221 Hayes Hall, Bowling Green, OH 43403 USA; 2 Faculty of Public and Allied Health, Bowling Green State University, 111 Health and Human Services Building, Bowling Green, OH 43403 USA; 3 Faculty of Applied Statistics and Operations Research, Bowling Green State University, 1001 E Wooster Street, Maurer Center 241J, Bowling Green, OH 43403 USA; 4 Faculty of Kinesiology and Community Health, University of Illinois at Urbana-Champaign, 1206 Fourth Street, IL 61820 Champaign, USA

**Keywords:** Drug overdose, Heroin-related incident, Clustering, Geospatial analysis, Emergency medical service response (EMS), Socioeconomic factors

## Abstract

**Background:**

Drug overdose is one of the top leading causes of accidental death in the U.S., largely due to the opioid epidemic. Although the opioid epidemic is a nationwide issue, it has not affected the nation uniformly.

**Methods:**

We combined multiple data sources, including emergency medical service response, American Community Survey data, and health facilities datasets to analyze distributions of heroin-related overdose incidents in Cincinnati, Ohio at the census block group level. The Ripley’s K function and the local Moran’s I statistics were performed to examine geographic variation patterns in heroin-related overdose incidents within the study area. Then, conditional cluster maps were plotted to examine a relationship between heroin-related incident rates and sociodemographic characteristics of areas as well as the resources for opioid use disorder treatment.

**Results:**

The global spatial analysis indicated that there was a clustered pattern of heroin-related overdose incident rates at every distance across the study area. The univariate local spatial analysis identified 7 hot spot clusters, 27 cold spot clusters, and 1 outlier cluster. Conditional cluster maps showed characteristics of neighborhoods with high heroin overdose rates, such as a higher crime rate, a high percentage of the male, a high poverty level, a lower education level, and a lower income level. The hot spots in the Southwest areas of Cincinnati had longer distances to opioid treatment programs and buprenorphine prescribing physicians than the median, while the hot spots in the South-Central areas of the city had shorter distances to those health resources.

**Conclusions:**

Our study showed that the opioid epidemic disproportionately affected Cincinnati. Multi-phased spatial clustering models based on various data sources can be useful to identify areas that require more policy attention and targeted interventions to alleviate high heroin-related overdose rates.

**Supplementary Information:**

The online version contains supplementary material available at 10.1186/s12889-022-13557-3.

## Background

Drug overdose is one of the top leading causes of accidental death in the U.S., largely due to the opioid epidemic [1]. Since 1999, more than 760,000 people have died from drug overdose, and the number of opioid-involved overdose deaths has increased over six times across the U.S. [2]. Drug overdose deaths involving synthetic opioids other than methadone have continued to rise since 2014 with more than 36,359 overdose deaths reported in 2019, which accounted for 72.9% of opioid-involved overdose deaths [3]. Furthermore, over the last 10 years, drug overdose deaths involving heroin rose more than seven times, although the trend has gone down since 2016 [2, 3]. Although the opioid epidemic is a nationwide issue, it has not affected the nation uniformly [4]. For example, opioid-related mortality rates, especially from synthetic opioids, have increased more rapidly in the eastern part of the country compared to other regions [5]. Understanding geographic variability in the opioid epidemic may help identify areas that require more attention and develop targeted strategies to tackle the public health challenge.

Some studies showed opioid-related emergency department visits increased over time [6, 7]. A study that examined naloxone administrations obtained from emergency medical service (EMS) also showed the increased number of non-fatal opioid overdose events over time [8]. However, compared to opioid overdose deaths, relatively less attention has been paid to opioid-related incidents. Given that a non-fatal opioid overdose event is a significant predictor of a subsequent overdose death [9–11], it is important to understand the prevalence of the incidences and discuss potential interventions that can prevent future deaths. Our study focused on heroin-related incidents that used EMS.

Geospatial clustering has been used to analyze the geographic variation of phenomena [12], such as dengue infection prevalence [13], and spatial clusters of hand, foot, and mouth disease [14]. It allows the investigators to identify groups of spatial objects (i.e., clusters) that have similar characteristics and analyze patterns of the clusters (e.g., hot spots, cold spots). Several studies have applied geospatial clustering to examine opioid-related overdose incidents and deaths, such as identifying hot spots of the opioid epidemic [15, 16], understanding geographical patterns in ambulance runs and nonfatal overdose [17, 18], and determining potential target locations for publicly deployed naloxone kits [19].

We conducted a case study in Cincinnati, Ohio where the opioid epidemic has been a serious issue. Among U.S. states, Ohio had the highest number of opioid-involved overdose deaths per year from 2014 through 2017 [20]. Since 2009, in Ohio, drug overdose deaths have continued to increase except in 2018 [20]. Cincinnati, a large city in southwestern Ohio with a population of about 300,000 people, was one of 12 opioid hot spots in Ohio and had the highest per capita rates of a fatal overdose within the state between 2010 and 2017 [16]. In the city, heroin overdose incidences still remain high since heroin and prescription drugs have ravaged the area [21], and the growing prevalence of heroin abuse has led to other public health problems such as HIV and needle-borne diseases [22].

The objectives of this study are to 1) demonstrate how various sources of data can be used to analyze spatial patterns of heroin-related overdose incidents and 2) analyze demographic, socioeconomic, and contextual factors associated with the spatial patterns. Using EMS response data, American Community Survey (ACS) data, and health facilities datasets, we built multi-phased spatial clustering models and analyzed distributions of heroin-related overdose incidents at the census block group level in Cincinnati.

The rest of the paper is organized as follows. 2 section describes our approach, 7 section details the results, and 8 section provides discussions of our findings and conclusions of the paper, along with limitations and directions of future research.

## Methods

### Data collection and processing

We used the EMS dataset that includes all responses to heroin-related overdose incidents from the Cincinnati Fire Department [23] between January 1, 2015, and December 31, 2020. Each incident record contains incident location information, including geospatial information, location address, a classification of a neighborhood in the city, time of the incident, and disposition of incident response. Data records regarding incidents outside of the study area, without geospatial information, and with unassociated disposition codes were excluded from this study. Records from canceled calls or false alarms and duplicated records were also excluded.

To identify demographic and sociographic characteristics corresponding to the EMS dataset, we utilized the ACS data for 2015 – 2019 [24]. The dataset included demographic information for each census block group, such as the adult population size, the population groups by age, the ratio of gender, and the ratio of race/ethnicity. The ACS data also included socioeconomic characteristics of each census block group, such as education, income, and poverty. In addition, we used Homeland Infrastructure Foundation-Level Data (HIFLD) [25] and Substance Abuse and Mental Health Services Administration (SAMHSA) data [26] for 2015–2020 to obtain information about available healthcare services, such as hospitals, opioid treatment programs, and buprenorphine prescribing physicians, in each census block group. Using the SAMHSA data, we computed the distance from the center of each census block to each of the nearest neighborhood [26]. Furthermore, we included the crime rate data from the Cincinnati Police Department [27] between 2015 and 2020.

In the next phase, we mapped individual heroin-related incidents to census block groups in Cincinnati using the geocode information and computed the average number of incidents in each census block for five years. The number of incidents was adjusted for the size of the population of a census block group, and finally, heroin-related incidents per 1,000 adult population were used in the analysis. Using the same procedures, we computed the average crime rate per 1,000 adult population in each census block group level. The average heroin-related incidents and crime rate per 1,000 adult population were merged with the ACS, HIFLD, and SAMHSA datasets, which resulted in 22 variables.

### Geospatial analysis

Using the merged dataset, we first examined the spatial distribution of heroin-related incident rates using the Jenks natural breaks maps. The Jenks natural breaks maps use a nonlinear algorithm to create groups where within-group similarity is maximized, and between-group similarity is minimized [28]. The number of groups was determined by optimizing the goodness of variance fit.

We then performed a multi-phased geospatial analysis to examine geographic variation patterns in heroin-related overdose incidents within the study area. Our objective was to identify the clusters with high incident rates. The spatial clustering was performed at two levels: global and local clustering. The global spatial clustering was conducted using Ripley’s K function that tests if heroin-related overdose incidents occur randomly or are clustered within the whole study area [29, 30]. The local clusters were determined based on the local indicators of spatial analysis (LISA), using the Local Moral’s I tool in GeoDa software [31]. The following sections explain in more details about each analysis.

#### Global spatial analysis

The Ripley’s K function is a multi-distance spatial clustering method that describes the dispersed patterns of data points. The function is calculated at multiple distances, which shows how point-pattern distributions can change with scale and compares the observed and expected distributions of points around an index point within circles of various areas. Under the complete spatial randomness, the density of points is uniform. If the observed estimate of the K function falls above the theoretically expected envelope, it indicates that the data points are clustered at every distance in the study area. On the other hand, if the observed estimate of the K function is within the envelope, the data points exhibit complete spatial randomness. Furthermore, if the observed estimate of the K function is below the envelope, it means that the data points are dispersed within the area of interest.

Given the locations of all events within a defined study area, the estimated K(t) is the ratio of the number of neighboring events observed within a given distance of each event and the density of events, $\lambda$. The density can be estimated as $\widehat{\lambda }=N/A$, where *N* is the observed number of points and *A* is the area of the study region. The estimated K(t) function [29] is shown in Eq. 1:

1 $${\varvec{K}}\left({\varvec{t}}\right)={\widehat{{\varvec{\lambda}}}}^{-1}\sum_{{\varvec{i}}=1}^{{\varvec{n}}}\sum_{{\varvec{j}}\ne {\varvec{i}}}^{{\varvec{n}}}{{\varvec{w}}{({\varvec{l}}}_{{\varvec{i}}},{{\varvec{l}}}_{{\varvec{j}}})}^{-1}\frac{{\varvec{I}}\left({{\varvec{d}}}_{{\varvec{i}}{\varvec{j}}}<{\varvec{t}}\right)}{{\varvec{N}}},$$

where *t* is the radius of a test circle, ${d}_{ij}$ is the distance between the *i-*th and *j-*th points, and $I(x)$ is an indicator function. The weight function, $w{(l}_{i},{l}_{j})$, corrects for edge effect. If the distance between ${l}_{i}$ and ${l}_{j}$ is less than or equal to *t* (i.e., the circle which centered at ${l}_{i}$ and passed through ${l}_{j}$ is inside the study area), the weight will be equal to 1. If part of the circle falls outside the study area, then the weight is equal to the proportion of the circumference of that circle that falls in the study area.

To calculate the Ripley’s K function, we pre-processed the geocode information of the heroin-related incidents by assigning the World Geodetic System (WGS84) as its reference coordinate system and projecting it to Universal Transverse Mercator (UTM). The pre-processed heroin-related incidents were converted to point pattern objects. With the converted point pattern objects, we performed the Ripley’s K analysis. To calculate confidence intervals for complete spatial randomness of heroin-related incidents within the study area, we conducted a Monte Carlo simulation [29]. Since the simulation outcomes of more than 999 replications remain identical, we set our maximum replications of the simulation at 999 and generated the confidence interval of the outcome at $\alpha =0.01$.

#### Local spatial analysis

Since the global spatial clustering analysis yields only one statistic to describe the overall point pattern across the whole study area, it does not identify where the local clusters or spatial outliers are. To determine which areas are similar or different from the neighboring areas, we performed a local spatial analysis. Local indicators of spatial association (LISA) decompose global indicators into the contributions of individual observations to identify local cluster patterns or spatial outliers [32]. LISA statistics satisfy two requirements: 1) for each observation they provide a statistic with an assessment of the significance of the grouping of similar values around this observation, and 2) they establish a proportional relationship between the sum of the local indices on all observations and a corresponding global index.

In LISA, spatial autocorrelation for each location with its neighbors is evaluated in five categories: high-high, low-low, low–high, high-low, and not significant. Positive spatial autocorrelation is captured in areas close together that have similar values. In this study, an area evaluated as high-high indicates it has high heroin-related overdose incident rates and its neighboring areas also have high heroin-related incident rates (hot spots), whereas an area evaluated as low-low indicates it has low heroin-related overdose incidents rates and its neighboring areas also have low rates (cold spots). On the other hand, negative spatial autocorrelation occurs when dissimilar values are shown between an area and its neighbors (e.g., low–high and high-low).

The local Moran’s I is a widely used LISA statistic which describes spatial clustering of observations in high or low values. For each observation *i*, the local Moran’s I equation [32] is shown in Eq. 2:

2 $${{\varvec{I}}}_{{\varvec{i}}}=\frac{{{\varvec{z}}}_{{\varvec{i}}}}{{{\varvec{m}}}_{2}}\sum_{{\varvec{j}}}{{\varvec{w}}}_{{\varvec{i}}{\varvec{j}}}{{\varvec{z}}}_{{\varvec{j}}};\boldsymbol{ }{{\varvec{m}}}_{2}=\frac{\sum_{{\varvec{i}}}{{{\varvec{z}}}_{{\varvec{i}}}}^{2}}{{\varvec{n}}},$$

where ${z}_{i}$ is the deviation from the mean (i.e., the difference between the actual value of $i$ and the mean), ${z}_{j}$ is the deviation from the mean for a neighboring area $j$, ${m}_{2}$ is the sample variance, ${w}_{ij}$ is the spatial weight for the pair of observations $i$ and $j$, and $n$ is the number of observations. That is, the Local Moran’s I statistic is computed as the product of a value at location $i$ by its weighted sum of the values at neighboring locations, where the product is standardized by the sample variance of all the observations.

We performed a univariate Local Moran’s I analysis for heroin-related incident rates using a software called GeoDa [31]. After identifying local spatial clusters, we plotted LISA conditional cluster maps to examine if there is a relationship between heroin-related incident rates and sociodemographic characteristics of areas. We considered five characteristics, including the proportion of males, the proportion of people below the poverty level, the proportion of people with at least a college level education, per capita income, and the crime rate. These factors were chosen because they have been known to be associated with opioid incidents and mortality [16, 33, 34]. To determine distributions of hot zones for a combination of two variables, we calculated a conditional probability of hot zones for one variable given a level (low vs. high) of the other variable. For example, we computed the conditional probability of hot zones for poverty when the education level was low and high. Similarly, we computed the conditional probability of hot zones for education when the poverty level was low and high.

In addition, we examined the relationships between heroin-related incident rates and the accessibility to three healthcare facilities, which are hospitals, opioid treatment programs, and buprenorphine prescribing physicians. Examination was completed separately using a queen contiguity-based spatial weights matrix in GeoDa.

For all the local spatial methods, we set the statistical significance level to 0.01, and the number of simulations to 9,999 since trying more than 9,999 permutations given a significance level of 0.01 generated identical outcomes.

## Results

Between January 1, 2015, and December 31, 2020, there were 542,136 incidents in total within the study area, of which 10,917 were heroin-related incidents. After removing the irrelevant and duplicated incidents, we used a total of 8,767 heroin-related incidents within the 280 census block groups in Cincinnati, Ohio. Table 1 shows the descriptive statistics of the data used in our study and the distribution of heroin-related incidents.

**Table 1:** Descriptive statistics of the data

Variables	Median (IQR)
Population, *n*	978.50 (665.0)
Population density per sq mi, *n*	5,269.70 (4458.16)
Sex	
Male, %	48.88 (10.21)
Age (years)	
18–24, %	7.98 (7.50)
25–34, %	17.61 (12.80)
35–44, %	11.30 (7.26)
45–55, %	10.61 (7.07)
55–64, %	11.75 (8.08)
65 + , %	10.80 (9.76)
Race/Ethnicity	
Non-Hispanic White, %	50.13 (53.87)
Non-Hispanic Black, %	37.63 (55.77)
Hispanic, %	1.41 (4.27)
Education level	
Less than High School, %	10.90 (13.05)
High School, %	54.62 (26.92)
Bachelor or Higher, %	29.13 (36.59)
Poverty, %	22.25 (29.51)
Per Capita Income, $	24,672.50 (21,465.25)
Accessibility to Health Facilities	
Distance to Hospitals, 10 miles	1.57 (1.55)
Distance to Buprenorphine, 10 miles	0.61 (0.67)
Distance to OTP*, 10 miles	2.14 (2.58)
Crime Rate per 1000 population	7.65 (8.71)
Heroin Incident Rate per 1000 population	0.26 (0.53)

^*^ Opioid Treatment Programs

**Fig. 1:**
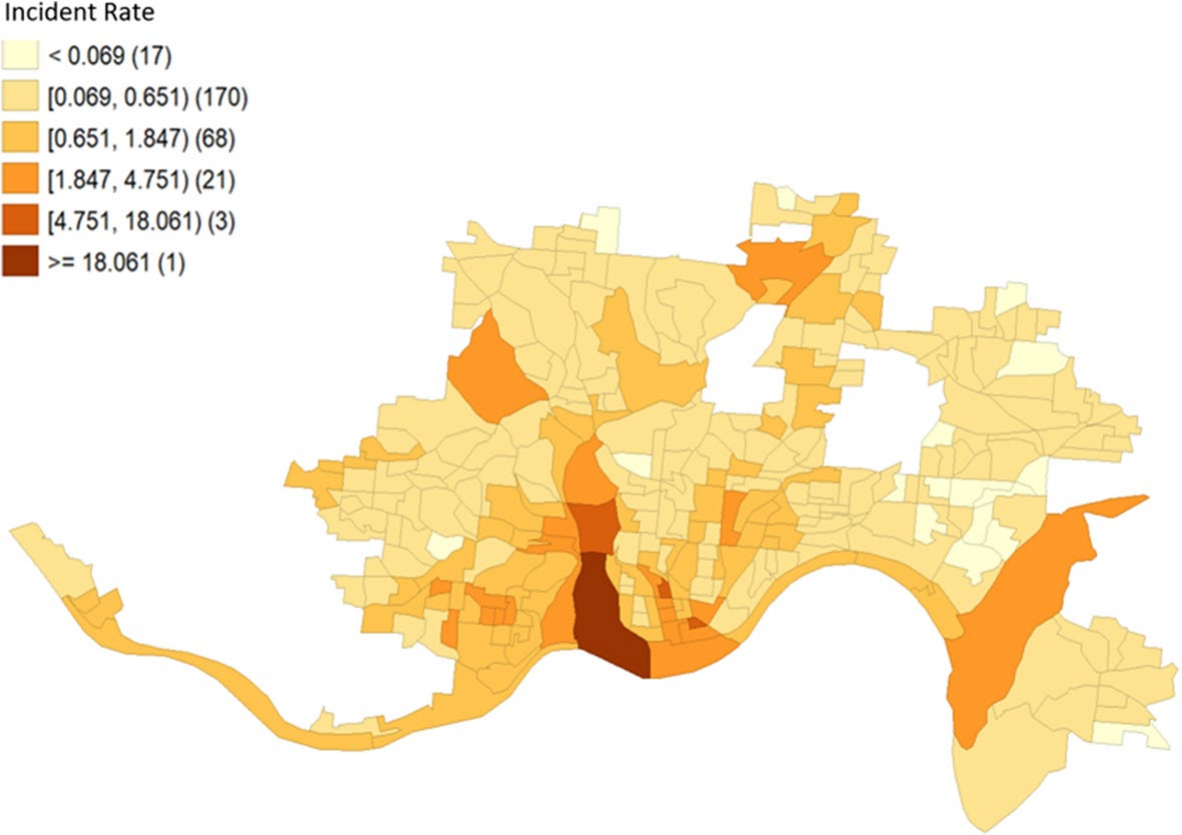
Distribution of heroin-related overdose incident rates by census block groups in Cincinnati, Ohio, from January 2015 to December 2020

Figure 1 shows six clusters of census block groups for heroin-related incident rates as determined by the Jenks natural breaks method, with darker colors indicating higher rates. The results indicated that there were geographical differences in heroin incident rates in Cincinnati: the blocks in the Southwest part of the city had high heroin-related incident rates while those in the Northeast part had low incident rates.

**Fig. 2:**
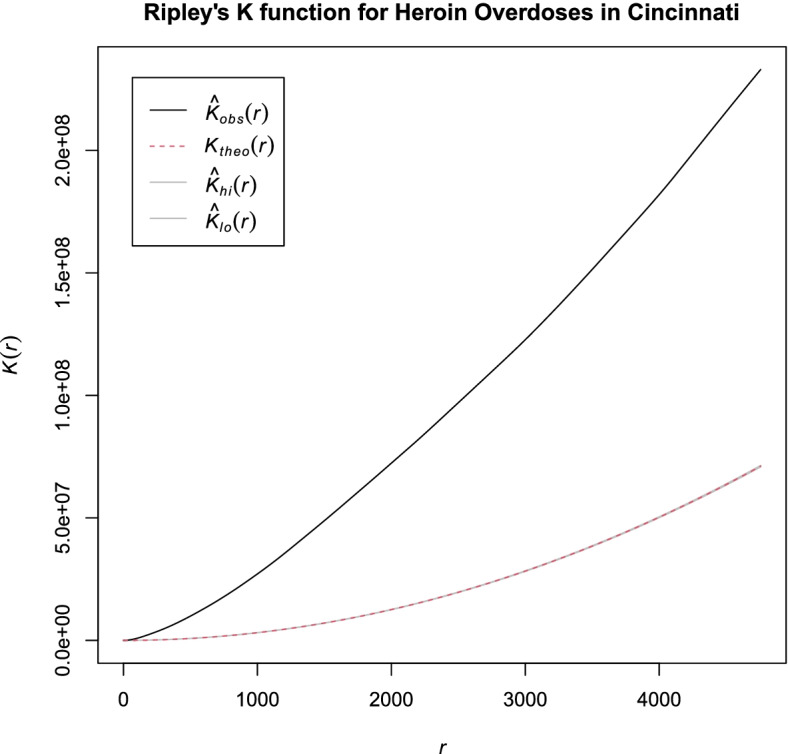
Ripley’s K functions measuring overall spatial clustering across Cincinnati, Ohio for heroin-related incident rates. (Obs: observed; Theo: theoretical)

Figure 2 shows the result of the global spatial analysis. Since the estimate of the observed K function (solid black curve) falls above the theoretical K generated by the Monte Carlo estimate (dashed red curve), we can conclude that there was a clustered pattern of heroin-related overdose incident rates at every distance across the study area.

**Fig. 3:**
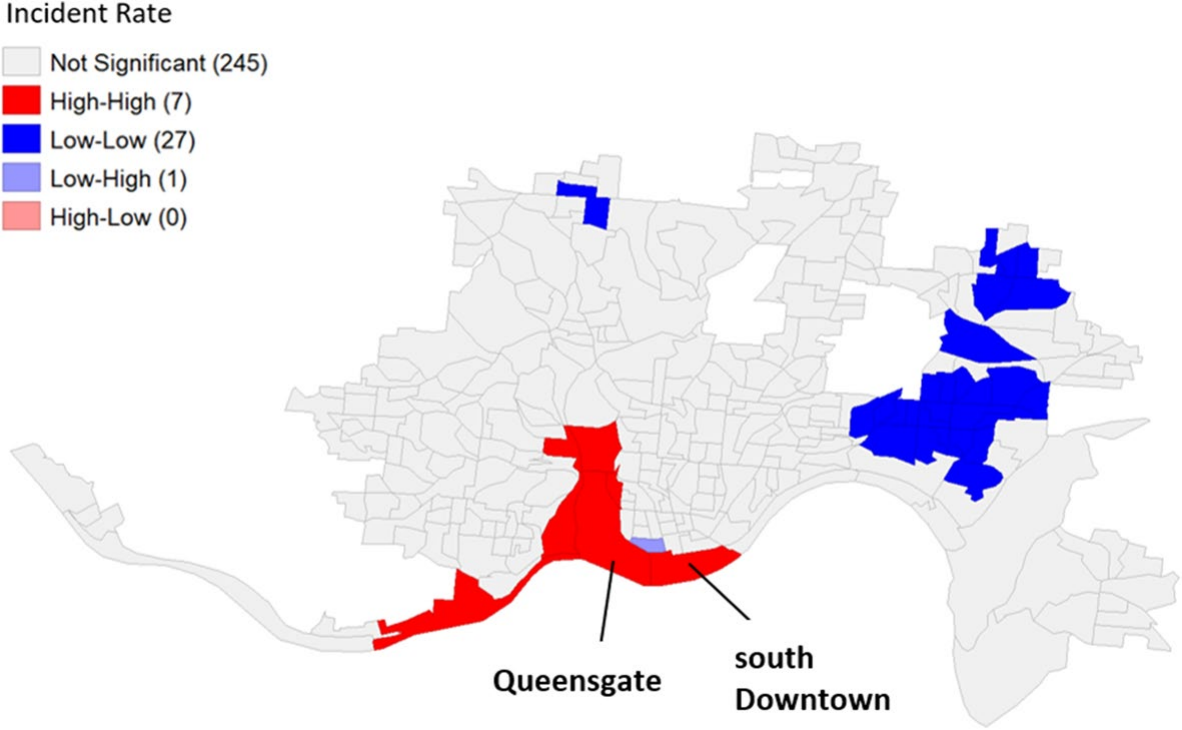
Univariate LISA cluster map identifying significant local clusters of heroin-related incident rates, Cincinnati, Ohio, 2015–2020: High-High: high incident rate within a block and high incident rate of neighboring blocks, Low-Low: low incident

The univariate LISA cluster map (Fig. 3) shows four types of spatial association determined based on heroin-related overdose incident rate within a block and its neighboring blocks: High-High, Low-Low, Low–High, and High-Low. If a block has a high heroin incident rate with neighboring blocks of high heroin incident rates, it was determined as a high-high cluster, also known as a hot spot. In the same scheme, if a block shows a low heroin incident rate with neighboring blocks of low heroin incident rates, it was classified as a low-low cluster, also known as a cold spot. On the other hand, if a block has a low incident rate with high heroin incident rate neighbors, it is classified as a low–high outlier. Likewise, if a high heroin incident rate block has low heroin incident rate neighbors, it is classified as a high-low outlier. Our analysis identified seven high-high clusters, 27 low-low clusters, one low–high spatial outlier, and zero high-low spatial outliers within the study area. The remaining 245 blocks did not have significant associations between a block and its neighbors in terms of heroin-related incident rates, which means that the heroin-related incident rates in these blocks were randomly distributed.

**Fig. 4:**
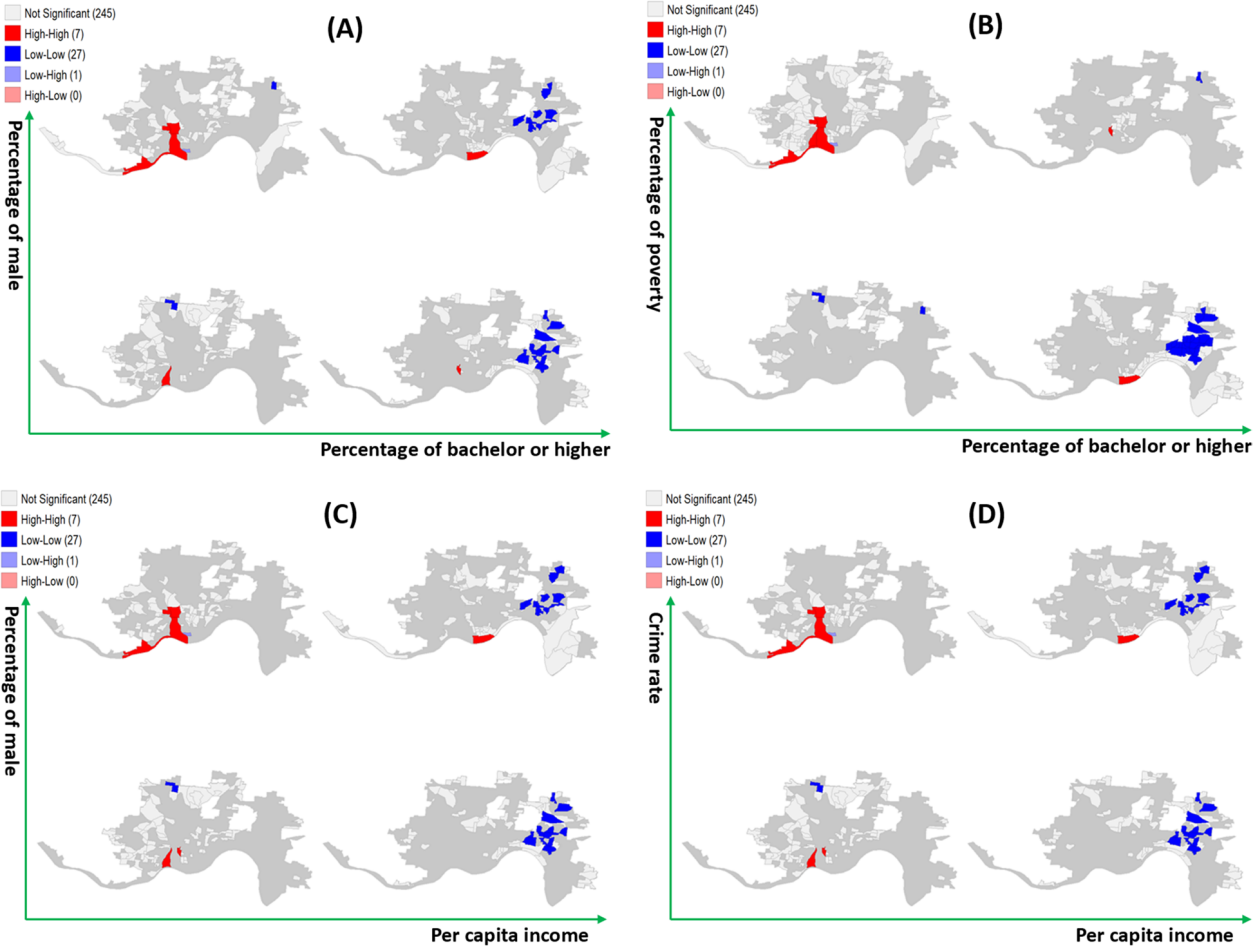
LISA conditional cluster maps for combinations of two variables

Figure 4 shows the LISA conditional cluster maps for heroin incident rates based on five socio-demographic characteristics of areas. We included combinations that had conditional probabilities higher than 0.6 for both variables at a certain level, and only four combinations shown in Fig. 4 met the criteria. The conditional probabilities of hot zones for these combinations are shown in Tables A1 – A4 in Appendix. The two-by-two micro maps were plotted based on median values of two of the five characteristics. For example, in Fig. 4 (A) the top right map shows clusters in regions with a higher percentage of males and a greater percentage of higher education than their medians, while the clusters in the bottom left map are regions with a lower percentage of male and a lower percentage of higher education. Similarly, Fig. 4 (B) shows the distributions of heroin incident rates by the education level and poverty level, 4 (C) shows the distributions by the percentage of male and income level, and 4 (D) shows the distributions by the crime rate and income level.

As shown in Fig. 4 (A), 71% of the hot spots were located in areas with lower education level and higher male percentage. On the other hand, 92% of the cold spots were located in areas with higher education and 52% of the cold spots were in areas with a lower male percentage. In Fig. 4 (B), 71% of hot spots were identified in areas with lower education and 86% of the hot spots were in areas with higher poverty level, which indicates hot spots tended to locate in areas with low education and high poverty level. On the other hand, 88% of the cold spots were located in areas with higher education and low poverty level. In Fig. 4 (C), 86% of the hot spots were identified in areas with lower income level and 71% of hot spots were in areas with a higher male percentage, which indicates hot spots tended to locate in areas with low-income level and high proportion of male population. On the other hand, 96% of the cold spots were located in areas with higher income level and 48% of the cold spots were in areas with lower male percentage and higher income level. In Fig. 4 (D), 86% of hot spots were identified in areas with lower income level and 100% of the hot spots were in areas with higher crime rate, which indicates hot spots tended to locate in areas with low-income level and high crime rate. On the other hand, 88% of the cold spots were located in areas with higher income level and low crime rate.

**Fig. 5:**
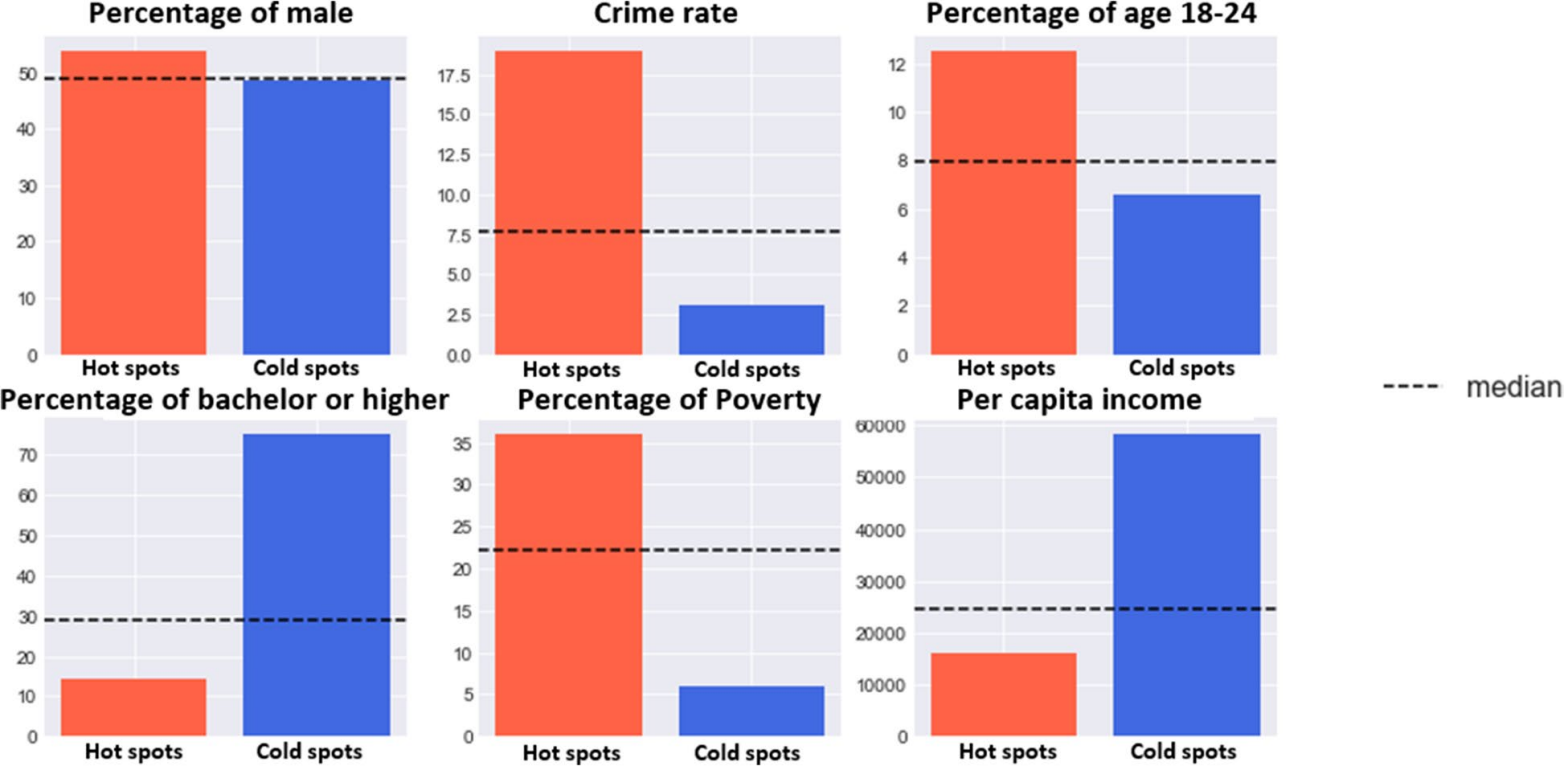
Socio-demographic characteristics of hot spots and cold spots identified from LISA

Figure 5 compares the socio-demographic characteristics of hot spots and cold spots identified from the conditional LISA maps within the study area, in which a dashed line represents the median of each variable. The results indicated that hot spots can be found in areas with a higher crime rate, a higher percentage of male population, a higher poverty level, a lower percentage of the population with higher education, and a lower per capita income.

**Fig. 6:**
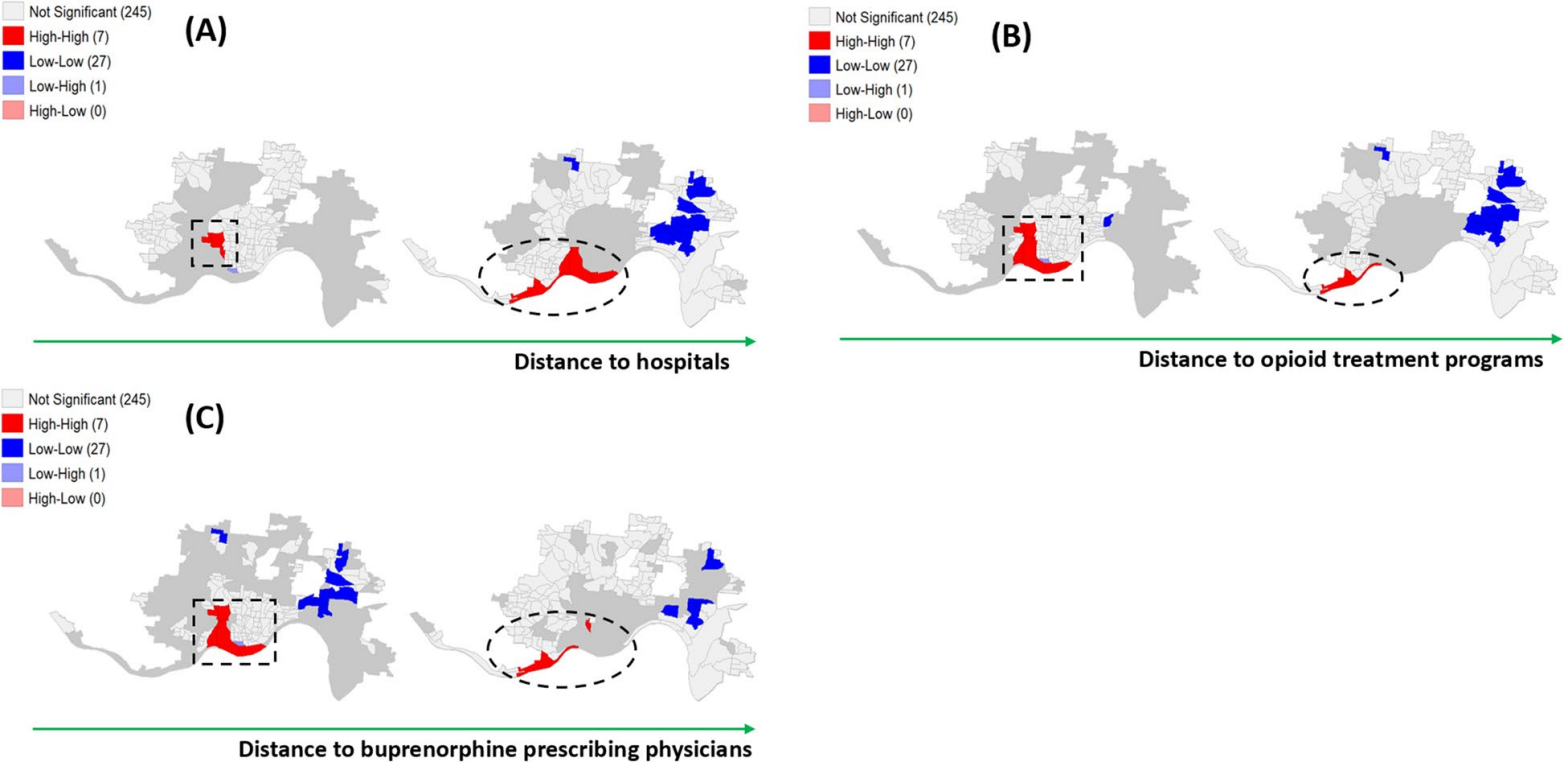
LISA conditional cluster maps of the distance to (**A**) hospitals, (**B**) the distance to Opioid Treatment Programs, (**C**) the distance to buprenorphine prescribing physicians

Figure 6 shows LISA conditional cluster maps of the accessibility to healthcare facilities such as hospitals (A), opioid treatment programs (B), and buprenorphine prescribing physicians (C). The two micro maps were plotted for each health facility based on its median distance from each census block group center. Figure 6 (A) shows that 57% of the hot spots were in areas with a longer distance to hospitals than its median. On the other hand, Fig. 6 (B) shows that 86% of the hot spots were in areas with a shorter distance to opioid treatment programs. Figure 6 (C) shows that 71% of the hot spots were in areas with a shorter distance to buprenorphine prescribing physicians. The hot spots in the Southwest part of Cincinnati (shown in dotted ellipses) had high heroin-related incident rates and longer distances to all three health facilities, while the hot spots of the South-Central part of the city (shown in dotted squares) had high heroin incident rates but shorter distances to opioid treatment programs and buprenorphine prescribing physicians.

## Discussion

This study showed how various data sources can be combined and used to examine the spatial patterns of heroin-related overdose incidents in Cincinnati, Ohio at the census block group level. We used EMS data to examine geographical distributions of heroin-related overdose incidents. This data is useful to understand real-time drug overdose cases that require emergency care. In addition to EMS data, we used ACS data, crime data, and healthcare facility data to understand demographic, socioeconomic, and contextual factors that may be associated with heroin-related incidents.

There have been studies that used EMS data to examine geographical patterns of opioid overdose events. Pesarsick et al. [18] and Dworkis et al. [19] utilized naloxone administration information in EMS data to identify local clusters for opioid overdose-related EMS runs. Li et al. used heroin-related overdose incident information from the EMS data within Cincinnati, Ohio to identify hot spots and potential target sites for naloxone kits where they are most needed in a community. They also focused on identifying significant predictors of the incidents using a Poisson spatiotemporal regression model. Other commonly used data sources for public health surveillance include medical examiner data [35], hospital electronic health record data [36], and emergency department visit data [37].

Our spatial analysis showed that the opioid epidemic disproportionately affected Cincinnati. For example, a heroin-related incident rate was concentrated in the Southwest part of the city, while the rate was low on the East side. This geographic disparity has been found for other outcomes related to drug overdose events. According to the City of Cincinnati Community Health Assessment [38], the southern part of the city had high rates of overdose visits to hospitals and emergency departments. Also, southern counties of Ohio have had high opioid-related overdose death rates. In particular, Hamilton County where Cincinnati is located had the third-highest number of overdose deaths in 2019 [39]. Our study investigated spatial patterns of heroin overdose incidents at a block group level, the smallest geographic unit for which the US Census Bureau publishes demographic and socioeconomic data, while other research regarding overdose mortality has been done at a larger geographic area level such as county [33, 34, 40]. A geographical analysis based on a finer-grained spatial unit may help capture more subtle but important patterns that might be missed when using larger spatial units.

In addition to the variability in heroin-related incident rates across Cincinnati, our study found demographic and socioeconomic characteristics in areas with high opioid overdose rates [15, 41, 42]. They include neighborhoods with a higher crime rate, a higher percentage of the male and younger age group (18–24) population, a lower education level, and a lower income level than the average of the city for each factor. These results are consistent with findings from other studies that analyzed the spatial and/or temporal characteristics of the opioid epidemic. Demographic factors (e.g., age, gender, and race) and socioeconomic factors (e.g., education and income) were significantly associated with the risk of opioid overdose incidents or deaths. For example, Hernandez et al. [16] found that the white male population aged 30–39 had the highest number of deaths due to prescription opioid abuse, followed by Black males aged 35–44. Amundsen et al. [34] found that drug-related deaths were more common in the population of a younger age group (15–44), the lowest level of education, and those not participating in the workforce. Li et al. [15] identified a positive association between the number of heroin-related incidents and features of the built environment, the proportion of the male population, the population aged 35–49 years, and a negative association between the number of heroin-related incidents and the proportion of the population with a bachelor's degree or higher, median household income, and the number of fast-food restaurants. Moreover, other studies [33, 43] found criminal justice involvement as one of the main risk factors associated with high opioid overdose rate.

Queensgate, in which the heroin-related incident rate was the highest among all the block groups in Cincinnati, showed distinct characteristics from other hot spots identified in this study. For example, this area had a much lower percentage of the white population and an extremely higher crime rate and male population. Queensgate mostly consists of industrial and commercial warehouses, and almost 100 percent of residents rent their homes. The level of urbanization and residential stability in a city may be an additional risk factor for opioid overdoses and related outcomes. Some studies found that people who rented were at an increased risk of fatal opioid overdose compared to those who owned a house [44, 45].

We also examined the association between the heroin-related incident rates and the access to healthcare resources for opioid use disorder (OUD) treatment such as hospitals, opioid treatment programs, and buprenorphine providers within census block groups. We hypothesized that hot spots have limited access to those facilities, and this was true in some areas. For example, distances from the center of a block group to the closest hospitals, opioid treatment programs, and buprenorphine providers were longer in the southeast area of the city than the average distances to each of the resources. However, contrary to our hypothesis, most of the hot spots had a shorter distance to the resources for OUD treatment compared to the average distances to each of the resources. For example, the south-central area of the city has a high heroin-related overdose incident rate but a shorter distance to the resources. Maxim et al. [46] found similar results about the geo-spatial correlation between the location of drug users and recovery houses. In their study, about 70% of reported overdose incidents occurred within 500 m of recovery houses. On the other hand, McLuckie et al. [47] found that rural counties in Illinois with high OUD rates had limited OUD-related services by their local health departments. This is a different finding from our study, which may be due to different study areas, such as rural versus metropolitan areas. Further investigation is needed to determine the relationship between opioid overdoses and the availability of resources.

Findings from geospatial analyses may serve as a basis for developing potential public health strategies that can alleviate high heroin-related overdose rates. For example, we plotted hot zones and cold zones by the accessibility to healthcare facilities to OUD. Hot zones that have limited accessibility to healthcare resources for OUD (i.e., long distance to the closest facility) would require different approaches than hot zones that have available resources close to the areas. In this study, Southwest areas of the city (shown in dotted ellipses in Fig. 6) had high heroin-related overdose incident rates while accessibility to healthcare resources for opioid use disorder treatment was limited. This kind of areas may need to consider increasing the number of buprenorphine providers or opioid treatment programs and providing support to utilize services in nearby locations. Some of the South-Central areas of the city (shown in dotted squares in Fig. 6) also had high heroin-related overdose incident rates but had available resources for OUD within a shorter distance. This kind of areas may need to evaluate the utilization of the services they currently provide and identify strategies to promote the utilization if it is low. To reduce the heroin-related incidence rate across all hot spots in the city, it would be important to understand controllable risk factors and develop targeted interventions for specific areas. Further analyses that consider associations between key characteristics will better inform effective and targeted interventions for different areas.

Despite our findings, this study has a few limitations. First, diagnoses (e.g., opioid overdose) in the EMS data may be different from a final diagnosis determined by a physician in a hospital. That means, the heroin-related incident rates captured through the EMS data might be under or overestimated than the actual rate. Also, heroin-related incidents used in this study do not represent the overall incidence rates that occurred in the city. Cases seen in various care settings, neither through the EMS and nor addressed at all, may have different geographical patterns. Another limitation is related to the estimation of a distance to the closet buprenorphine prescribing physicians. We used publicly available treatment locator data to identify buprenorphine prescribing physicians. However, we might not include buprenorphine-waivered clinicians because they did not consent to be on the buprenorphine list and therefore were not shown in the data. In addition, we might have included buprenorphine-waivered providers who were on the buprenorphine list but no longer actively prescribed buprenorphine. Despite these limitations, studying local EMS data is still critical for timely and targeted public health intervention in that it is the first to respond to medical emergencies.

Future studies will generalize our multi-phased geospatial analysis to other cities that are highly associated with the heroin overdose epidemic. The current study can be improved by linking EMS data with other related data sources, such as naloxone administration by first responders, to better understand geospatial characteristics of a heroin overdose. In addition, combining spatiotemporal disparities with machine learning models may help identify patterns at varying spatial and temporal scales more accurately.

## Supplementary Information

**Additional file 1.**

## Data Availability

The data sets generated and/or analyzed during the current study are available in the below repositories: Cincinnati Fire Incidents (CAD) (including EMS: ALS/BLS) Safety: https://data.cincinnati-oh.gov/Safety/Cincinnati-Fire-Incidents-CAD-including-EMS-ALS-BL/vnsz-a3wp American Community Survey (ACS): https://data.census.gov/cedsci/ Homeland Infrastructure Foundation-Level Data (HIFLD): https://hifld-geoplatform.opendata.arcgis.com/ SAMHSA. OTP Directory: https://dpt2.samhsa.gov/treatment/directory.aspx PDI (Police Data Initiative) Crime Incidents: https://data.cincinnati-oh.gov/Safety/PDI-Police-Data-Initiative-Crime-Incidents/k59e-2pvf

## Abbreviations

EMS: Emergency Medical Service
ACS: American Community Survey
HIFLD: Homeland Infrastructure Foundation-Level Data
SAMHSA: Substance Abuse and Mental Health Services Administration
UTM: Universal Transverse Mercator
LISA: Local Indicators of Spatial Association
MCE: Monte Carlo estimates
OUD: Opioid Use Disorder
