# Supplementary material for: Spatial clustering of heroin-related overdose incidents: a case study in Cincinnati, Ohio
Source: BMC Public Health. 2022 Jun 25;22:1253. doi: 10.1186/s12889-022-13557-3 (PMC9233379; doi:10.1186/s12889-022-13557-3)
Supplement: Supplementary file 1 — Additional file 1. [file 12889_2022_13557_MOESM1_ESM.docx]

**Appendix**

Figure A1 (A) – (D) shows the LISA conditional cluster maps for heroin incident rates based on five socio-demographic characteristics of areas. Tables A1 –A4 show the conditional probabilities of hot zones for the combinations of the characteristics.

**
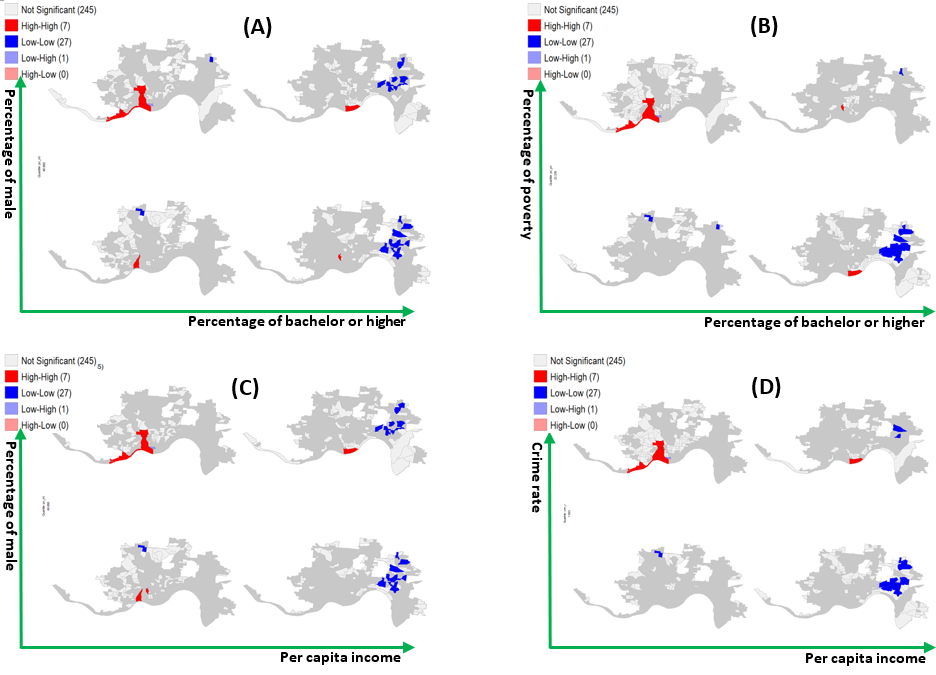
**

**Figure A1. LISA conditional cluster maps for combinations of two variables.**

Table A1.Conditional probabilities of hot zones for education and the percentage of male (Figure A1(A))

|  | Low education level | High education level | **Education total** |
| --- | --- | --- | --- |
| Low percentage of male | 0.14 | 0.15 | **0.29** |
| High percentage of male | 0.57 | 0.14 | **0.71** |
| **Male total** | **0.71** | **0.29** | **1** |

Table A2. Conditional probabilities of hot zones for education and poverty level (Figure A1(B))

|  | Low education level | High education level | **Education total** |
| --- | --- | --- | --- |
| Low poverty level | 0.0 | 0.14 | **0.14** |
| High poverty level | 0.71 | 0.15 | **0.86** |
| **Poverty total** | **0.71** | **0.29** | **1** |

Table A3. Conditional probabilities of hot zones for income and the percentage of male (Figure A1(C))

|  | Low income | High income | **Income total** |
| --- | --- | --- | --- |
| Low percentage of male | 0.29 | 0.0 | **0.29** |
| High percentage of male | 0.57 | 0.14 | **0.71** |
| **Male total** | **0.86** | **0.14** | **1** |

Table A4. Conditional probabilities of hot zones for income and crime rate (Figure A1(D))

|  | Low income | High income | **Income total** |
| --- | --- | --- | --- |
| Low crime rate | 0.0 | 0.0 | **0** |
| High crime | 0.86 | 0.14 | **1** |
| **Crime total** | **0.86** | **0.14** | **1** |
